# Lessons from Tau-Deficient Mice

**DOI:** 10.1155/2012/873270

**Published:** 2012-06-06

**Authors:** Yazi D. Ke, Alexandra K. Suchowerska, Julia van der Hoven, Dineeka M. De Silva, Christopher W. Wu, Janet van Eersel, Arne Ittner, Lars M. Ittner

**Affiliations:** Alzheimer's and Parkinson's Disease Laboratory, Brain and Mind Research Institute, The University of Sydney, 100 Mallett Street, Camperdown, NSW 2050, Australia

## Abstract

Both Alzheimer's disease (AD) and frontotemporal dementia (FTD) are characterized by the deposition of hyperphosphorylated forms of the microtubule-associated protein tau in neurons and/or glia. This unifying pathology led to the umbrella term “tauopathies” for these conditions, also emphasizing the central role of tau in AD and FTD. Generation of transgenic mouse models expressing human tau in the brain has contributed to the understanding of the pathomechanistic role of tau in disease. To reveal the physiological functions of tau *in vivo*, several knockout mouse strains with deletion of the tau-encoding *MAPT* gene have been established over the past decade, using different gene targeting constructs. Surprisingly, when initially introduced tau knockout mice presented with no overt phenotype or malformations. The number of publications using tau knockout mice has recently markedly increased, and both behavioural changes and motor deficits have been identified in aged mice of certain strains. Moreover, tau knockout mice have been instrumental in identifying novel functions of tau, both in cultured neurons and *in vivo*. Importantly, tau knockout mice have significantly contributed to the understanding of the pathophysiological interplay between A**β** and tau in AD. Here, we review the literature that involves tau knockout mice to summarize what we have learned so far from depleting tau *in vivo*.

## 1. Introduction

Alzheimer's disease (AD) is the most common form of dementia, characterized by a progressive decline of cognition due to synaptic and neuronal loss [1]. Despite intensive research into the cause of AD, there is no cure available to date, and current treatment options are limited to symptomatic relief [2]. This becomes even more alarming in the light of over 35 million AD patients worldwide, a number estimated to double by 2050 [3].

AD brains display two hallmark lesions upon autopsy: amyloid *β* (A*β*)-containing plaques and neurofibrillary tangles (NFTs). A*β* results from *β*- and *γ*-secretase-mediated proteolytic cleavage of the A*β*-precursor protein (APP) [4, 5]. NFTs are made up of hyperphosphorylated forms of the microtubule-associated protein tau [6]. In contrast to AD, FTD presents with tau pathology in the absence of an overt A*β* pathology. FTD is the second most prevalent form of dementia occurring before the age of 65 [7–9]. FTD describes a heterogeneous group of neurodegenerative disorders, including Pick's disease (PiD), frontotemporal dementia with Parkinsonism linked to chromosome 17 (FTDP-17), argyrophilic grain disease (AGD), corticobasal degeneration (CBD), and progressive supranuclear palsy (PSP). Sharing similar protein deposits, these disorders are characterized by a broad spectrum of clinical symptoms including behavioural changes, language abnormalities, and motor dysfunction (reviewed in [7, 9]). While in familial cases of AD (FAD), mutations were found in the APP- and the presenilin genes 1 and 2 [10], the latter being part of the *γ*-secretase complex [11], mutations in the MAPT gene were found in familial FTD [12, 13].

The tau protein has been discovered in 1975 as a protein with the ability to induce microtubule (MT) formation [14]. The tau-encoding gene *MAPT* is located on human chromosome 17q21 [15]. There are 6 tau isoforms, between 352 and 441 aminoacids in length, encoded by 11 exons in humans, with alternative splicing of exons 2, 3, and 10 [16]. They differ by either the presence or absence of up to two amino-terminal inserts (2N) and by containing either three (3R) or four (4R) MT-binding repeats (MTB). While 3R isoforms are predominant during embryonic brain development, the normal adult brain has approximately equal levels of 3R and 4R isoforms [17]. Changes in this ratio have been linked to the pathogenesis of tauopathies, with increased 4R levels in AD and high amounts of 3R tau in PiD [18]. For comparison, mice and rats express only three different 4R isoforms of tau, but lack 3R tau [19].

Tau is expressed predominantly in neurons, where it is enriched in axons. Tau is either bound to microtubules, the inner side of the plasma membrane, or is unbound [20]. Besides stabilizing microtubules, tau has been implicated in the regulation of motor-driven axonal transport [21, 22]. Other possible functions of tau include cellular signalling, neuronal development, neuroprotection, and apoptosis [3, 23]. Furthermore, we have shown that tau is also present in dendrites at low levels, where it is involved in postsynaptic scaffolding [3, 24].

In AD and FTD, tau becomes increasingly phosphorylated at both physiological and pathological sites (referred to as “hyperphosphorylated”) [25]. This aberrant phosphorylation detaches tau from microtubules, thereby probably compromising its microtubule-stabilising functions (loss of physiological function). At the same time, hyperphosphorylation of tau is the first step in the formation of toxic aggregates (gain of toxic function) and eventually of NFTs [26]. Hyperphosphorylated tau accumulates in the soma of neurons, giving rise to increased dendritic levels of tau [24, 27, 28]. However, there is good evidence that elevated levels of soluble tau already contribute to neuronal dysfunction prior to its deposition [29, 30], including for example, disruption of axonal transport [31–34] and impairment of mitochondrial function [35, 36].

The identification of pathogenic mutations in AD and FTD has led to the generation of multiple transgenic animal models that recapitulate important aspects of the human disease [37]. Transgenic mouse models, including those with human tau expression, have become the major *in vivo* tool in AD/FTD research (reviewed in [37]). In addition to tau overexpressing mouse strains, several tau knockout strains have been generated. Their contribution to the understanding of tau is reviewed in this paper.

## 2. Tau Knockout Mice

Expression of tau in cell lines resulted in elongated process formation, while tau reduction using antisense RNA suppressed axon elongation in cultured neurons [38]. Based on these findings in cells, tau depletion in mice was eagerly awaited with expectations of marked effects on neuronal systems *in vivo*. Surprisingly, four independently generated (conventional) tau knockout lines presented with no overt phenotype [39–42]. Only when aged did tau knockout mice develop behavioral impairments and motor deficits [43, 44].

**Figure 1:**
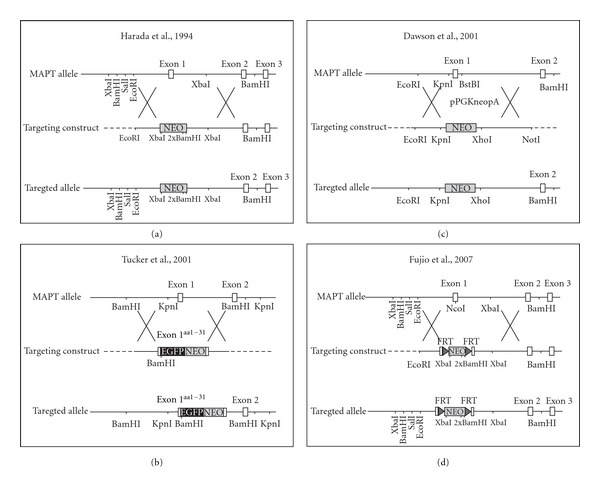
Homologous recombination strategies for the generation of different tau knockout mice reported by (a) Harada and collegues [39], (b) Tucker and colleagues [41], (c) Dawson and colleagues [40], and (d) Fujio and colleagues [42].

In 1994, Harada and colleagues reported the first tau knockout mouse line (Figure 1(a)) [39]. The mice are viable and macroscopically normal. While immunohistochemical analysis did not show changes, electron microscopical analysis revealed decreased microtubule density in axons, together with reduced cross-bridging between parallel microtubules, and between microtubules and the plasma membrane. Interestingly, neurons from this particular strain showed normal axonal development in culture [39, 45]. The lack of tau was associated with an up to 2 fold increase in the microtubule-associated protein 1A (MAP1A) expression in 7-day-old, and a 1.3 fold increase in adult tau knockout mice, possibly compensating for the absence of tau [39]. Although MAP1B levels were reportedly normal in this tau knockout line, cross-breeding of tau with MAP1B knockout mice exacerbated hypoplastic axon tracts, disorganized neuronal layering, and impaired maturation of primary neurons of MAP1B mice [45].

In 2001, two additional tau knockout lines have been published. Tucker and colleagues generated tau knockout mice by integrating GFP-encoding cDNA into exon 1 of *MAPT*, resulting in expression of a GFP fusion protein with aminoacids 1 to 31 of tau, together with deletion of endogenous tau expression (Figure 1(b)) [41]. While the original report did use heterozygote tau knockout mice to image GFP expressing neurons, as well as sorting neurons for *in vitro* analysis, tau function has not been studied. Nevertheless, this particular strain has been used in several subsequent studies to identify novel functions of tau [24, 46].

Dawson and colleagues generated tau knockout mice by homologous recombination replacing exon 1 with a neomycin expression cassette (Figure 1(c)) [40]. Again, these mice are viable and display no overt anomalies. Similar to previous tau knockout mice [39], MAP1A levels were approximately 2-fold increased at birth, but declined with brain maturation. In fact, MAP1A levels were similar in wildtype and tau knockout mice at 12 months of age [40]. Hence, MAP1A may compensate for loss of tau during early brain development, but not in the mature brain [39, 40, 42]. Contrary to previous studies, primary neurons obtained from this tau knockout strain showed slowed maturation with reduced neurite length throughout all developmental stages and reduced axon length of stage 3 neurons [40].

The most recent tau knockout mouse has been established by Fujio and colleagues in 2007 [42]. They introduced a neomycin cassette in reverse orientation flanked by flippase recognition targets (FRTs) into exon 1 of *MAPT* (Figure 1(d)). Similar to previous tau knockout mice, they are viable and show no overt anomalies. MAP1A levels were increased as previously reported for other tau knockout strains [40].

Taken together, in three of four independent tau knockout strains, MAP1A is increased around birth but not in adult brain (NB: MAP1A levels have not been determined in the fourth strain), suggesting early but not late compensation for loss of tau by MAP1A [39, 40, 42]. On the other hand, neuronal maturation has been examined in two of four tau knockout lines, with different results [39, 40, 47]. Here, analysis of additional lines may provide clarification. Differences between the different tau knockout strains may be explained by different genetic backgrounds used.

## 3. Deficits in Tau Knockout Mice

Neuronal dysfunction and neurodegeneration in tauopathies is due to the toxicity of pathologically modified tau and/or loss of physiological tau function [48]. While the reported tau knockout strains presented without overt phenotype when young [39–42], one-year-old mice of one tau knockout strain [39] showed muscle weakness in the wire-hanging test, reduced balancing in the rod-walking test, hyperactivity in new environments and impaired contextual fear conditioning [43]. Interestingly, muscle weakness was already detectable in heterozygote tau knockout mice. The spatial learning ability of this tau knockout strain, however, presented normal, when tested in the eight-arm radial maze and the Morris water maze [43]. Normal performance by 7- and 12-month-old tau knockout mice in the Morris water maze has since been independently confirmed in another strain [49, 50]. The latter strain also performed normal in the radial water arm maze and on the Rotarod at 12 months of age [50]. However, Lei and colleagues recently identified more complex motor deficits in the same tau knockout strain at 12 months of age, with increased turn time in the pole test, reduced performance on the Rotarod and decreased locomotion in the open field test [44]. These deficits were associated with reduced numbers of tyrosine hydroxylase-positive substantia nigra (SN) neurons [44]. Interestingly, mutant tau overexpression also results in a loss of SN neurons in a mouse model of FTD with Parkinsonism [33], suggesting that tau levels are critical for this neuronal population; however, the exact role of tau herein remains unknown. Taken together, tau knockout mice appear to develop motor deficits with increasing age, suggesting that loss of physiological tau function may contribute to the motor deficits observed in tauopathy patients. Further, tau knockout mice show no overt memory deficits in spatial memory tasks, which is consistent with sole tau pathology in humans, such as in FTD, where memory function is not or only mildly compromised.

Cantero and colleagues examined the local field potential from various cortical regions and hippocampus of tau knockout mice to determine if delayed axonal maturation of tau knockout neurons observed *in vitro* [40] could possibly affect neuronal circuit formation *in vivo* [51]. They found slower theta rhythms of the hippocampus and reduced gamma synchronization between cortical brain regions in tau knockout mice, which may suggest impaired circuit formation. To this end, the morphological correlates of these findings remain unknown, but dysfunctional neuronal networks have been implicated in the pathogenesis of AD [52]. Furthermore, tau knockout mice have abnormal sleep-wake cycle, with increased wakefulness periods and reduced nonrapid eye movements [53]. Taken together, tau may play a role in neuronal circuit formation.

## 4. Tau Knockout Mice with Human Tau Expression

Overexpression of human tau derived from a human P1-derived artificial chromosome (PAC) in mice results in expression of all 6 human tau isoforms in mouse brains in the absence of any neuropathology [54]. Interestingly, crossing of PAC tau transgenic mice with tau knockout mice results in accumulation of hyperphosphorylated tau and formation of sarkosyl-insoluble 3R, but not 4R tau as early as 2 months of age [46]. Furthermore, 12-month-old PAC tau transgenic mice on a tau knockout background present with memory deficits in the Morris water maze task and perturbed LTP formation [55]. These aged mice are further characterized by neuronal loss together with ventricle enlargement and reduced cortical thickness [56]. Similarly, depletion of mouse tau exacerbated tau pathology in transgenic mice expressing double mutant human tau [57]. Together, this suggests that endogenous mouse tau prevents pathological alteration of transgenic human tau. The underlying mechanisms remain, however, elusive.

Sennvik and colleagues generated knockin mice by inserting cDNA that encodes the longest human tau isoform (2N4R) into exon 1 of the* MAPT* gene [58]. Surprisingly, neuronal numbers were increased in the hippocampus of these mice, as a result of increased neurogenesis and neuronal survival during development. This is accompanied by an improved performance in the novel object recognition task. Similar to other tau-deficient strains [40], maturation of primary neurons is delayed in primary cultures from these human tau knockin mice, probably because expression of human tau is only detectable after 10 days *in vitro* (DIV). Interestingly, at 4 DIV, human tau knockin neurons show proliferative markers, which disappear at the onset of human tau expression at 10 DIV and neuronal maturation [58]. Although mechanistically unclear, this may suggest anti-proliferative and prodifferentiation effects of human 2N4R tau.

## 5. Gene and Protein Regulation in Tau Knockout Mice

Tau depletion is associated with a 2 fold increase in MAP1A levels in newborn mice, but they are reduced to normal levels thereafter [39, 40]. This possible early compensation of the loss of tau by MAP1A has been reported together with the first tau knockout mice [39]. Since then, tau knockout mice have been subjected to both educated guess approaches and unbiased screening methods to identify additional genes and proteins that are deregulated, to further understand tau's role *in vivo*.

Tau has been shown to interact with histone deacetylase 6 (HDAC6), a tubulin deacetylase, via the microtubule binding domain of tau and the SE14 domain of HDAC6 that mediates its enzymatic activity [59]. Perez and colleagues showed that tau, in particular aggregated tau, inhibits HDAC6 activity* in vitro*, together with reduced levels of acetylated tubulin in primary tau knockout neurons, suggesting that tau regulates HDAC6 activity [60]. Another class of proteins that forms complexes with tau is 14-3-3, a group of scaffolding proteins [61–63]. However, the absence of changes in levels and interaction with microtubules of different 14-3-3 isoforms in tau knockout mice suggest that the interaction between tau and 14-3-3 is rather only relevant under conditions with increased levels of unbound tau [42], given that the interaction involves the microtubule-binding domain of tau that is normally preoccupied with tubulin [61].

Unbiased gene expression analysis revealed several genes that are changed in tau knockout compared to wildtype mouse brains [47, 64]. Oyama and colleagues identified several mRNAs that are deregulated in brains of tau-deficient mice [64]. Of those, Gem GTPase, a regulator of Rho signaling and inducer of cellular process formation, was significantly increased in tau knockout brains. In cells, tau suppressed the activity of Gem GTPase via its microtubule-binding domain, suggesting that tau may be involved in regulating Gem GTPase downstream signaling [64]. In another mRNA screening, de Barreda and colleagues found increased BAF57 mRNA and protein levels in the hippocampus of tau knockout mice [47]. BAF57 interacts with coREST, which in turn activates the transcriptional repressor REST and consequently the expression of neuronal specific genes [65]. Here, tau may act as a nuclear regulator of gene expression. Accordingly, tau can be isolated from nuclei of hippocampal cells [47, 66].

Taken together, analysis of deregulated genes and proteins in tau knockout mice proves valuable for the identification of novel tau functions. Advanced screening methods, such as next-generation sequencing, may provide further insights into tau-dependent processes in the future.

## 6. Protection from A***β*** Pathology

According to the amyloid cascade theory, A*β* is upstream of tau pathology in the pathogenesis of AD [67]. This has been reproduced in mutant tau transgenic mouse models with NFT formation, by crossing them with A*β*-forming APP transgenic mice [68], or injecting A*β* into their brains [69], both resulting in increased NFT pathology. Interestingly, in 2002, Rapoport and colleagues provided the first evidence that tau is also needed for A*β* to cause its toxicity in neurons *in vitro*, as suggested by resistance of primary cultured neurons from tau knockout mice to A*β* exposure [70]. It was not until 2007, when Roberson and colleagues reproduced this finding *in vivo*, by crossing A*β*-forming APP transgenic mice, which display premature mortality and memory deficits, on a tau knockout background [49]. Both hetero- and homo-zygote tau deficiency rescued premature mortality and prevented memory deficits in APP transgenic mice. Mechanistically, this protection appeared to be conferred by reduced susceptibility to excitotoxicity in tau knockout mice [49]. Excitotoxicity describes a signalling cascade that is induced by overactivation of NMDA receptors (NMDARs) that results in neuronal damage and death, and excitotoxicity has been implicated as a pathomechanism underlying neurodegeneration induced by A*β* in AD [71]. We reproduced the protection from A*β*-induced premature mortality and memory deficits, using independent APP transgenic, and tau knockout mice, also showing that reduced susceptibility to excitotoxicity of the latter underlies this protection [24]. Furthermore, we used expression of a dominant-negative truncation mutant of tau to prevent deficits in APP transgenic mice. Together with the tau knockout mice, we were able to show for the first time that tau is critically involved in postsynaptic NMDAR downstream signalling, by localizing the Src kinase Fyn to dendrites, where it mediates coupling of NMDAR complexes to postsynaptic scaffolding proteins and therefore signalling cascades. Reduced postsynaptic Fyn levels in tau-deficient or truncated tau expressing mice results in uncoupling of NMDARs from excitotoxic downstream signalling and therefore prevention of A*β* mediated toxicity [3, 24]. Hence, A*β*, Fyn and tau may orchestrate neuronal damage in AD mouse models [24, 72, 73], suggesting a critical role of tau in the pathogenesis of AD [3]. This data is consistent with previous reports on the preventive effects of Fyn depletion and accelerating effects of Fyn expression on the deficits in APP transgenic mice [74, 75]. Further, supporting the protective effects of tau depletion, Shipton and colleagues showed recently that A*β*-mediated impairment of LTP in hippocampal slices of wildtype mice is prevented in tau knockout mice [76]. LTP formation *per se* was normal in tau knockout mice [76], consistent with normal excitatory postsynaptic potential (EPSP) recordings in two different tau knockout strains [24, 73]. To this end, while several studies showed protection from A*β* toxicity by knocking out tau [24, 49, 70, 77], one other study showed increased pathology in aged mice [50], possibly reflecting different effects based on the usage of different APP transgenic lines.

The exact downstream mechanisms involved in mediating protection from A*β* toxicity in tau knockout mice remains to be shown. These may include axonal transport, which is known to be regulated by tau [22]. While basal axonal transport rates are unaffected in tau knockout neurons [77, 78], impairment of axonal transport of mitochondria and TrkA-containing vesicles induced by A*β* is prevented in tau-deficient neurons [77]. Furthermore, increased A*β*-formation is associated with increased activity of GSK3*β*, a known tau kinase [79, 80]. GSK3*β* overexpression in the brain of mice results in degeneration of the dentate gyrus, but this is significantly ameliorated when crossed with tau-deficient mice [81]. Tau knockout neurons also show increased resistance to heat shock [82]. While Hsp70 levels were increased upon heat shock in both wildtype and tau knockout neurons, Akt phosphorylation was delayed together with a virtual absence of GSK3*β* activation. Whether Akt/GSK3*β* signalling plays a role in preventing A*β* toxicity in tau knockout mice remains to be shown. If other tau kinases or phosphatases are involved also remains to be shown. Furthermore, protection of tau knockout neurons may be conferred by nuclear tau [83].

The protection from A*β* toxicity in tau knockout mice seems to be rather specific, since it does not prevent deficits in models of several other neurodegenerative disorders. Accordingly, crossing of mutant SOD1 expressing mice, a model of amyotrophic lateral sclerosis (ALS), on a tau knockout background, does not prevent weight loss and death [73]. Furthermore, motor deficits of mouse models of Parkinson's disease (PD) are not prevented on a tau-deficient background [84]. The PD models used were striatal injection of 6-hydroxydopamine and transgenic expression of *α*-synuclein, neither of which showed improvement. Similarly, tau depletion does not protect from deficits induced by intracranial administration of prions [84]. Tau depletion is associated with silver positive spheroids in yet another APP transgenic mouse strain when aged [50]. Finally, knocking out tau even exacerbated the phenotype of NPC deficient mice, a model of Niemann-Pick disease type C, suggesting a role of tau in regulation of autophagy [85].

Taken together, tau reduction prevents mice from specific A*β*-mediated deficits, supporting a central role of tau in mediating A*β* toxicity in the early pathogenesis of AD. However, tau depletion does not generally prevent from neurodegenerative conditions, suggesting distinct mechanisms.

## 7. Concluding Remarks

To this end, tau knockout mice have significantly contributed to unraveling novel functions of tau under physiological condition and its role in disease. While key findings have been reproduced in independent tau-deficient strains, others, such as delayed axonal maturation [40], remain to be confirmed in alternative strains. Differences may also be due to the usage of different genetic backgrounds with possible confounding effects. Understanding the differences might contribute to a broader knowledge about the physiologic function of tau, which may be translated to understanding the mechanisms of tauopathies.
